# Advanced Metal–Organic Frameworks-Based Catalysts in Electrochemical Sensors

**DOI:** 10.3389/fchem.2022.881172

**Published:** 2022-03-31

**Authors:** Yana Chen, Zhiquan Yang, Huilin Hu, Xinchen Zhou, Feng You, Chu Yao, Fang Jun Liu, Peng Yu, Dan Wu, Junlong Yao, Ruofei Hu, Xueliang Jiang, Huan Yang

**Affiliations:** ^1^ Hubei Key Laboratory of Plasma Chemistry and Advanced Materials, School of Materials Science and Engineering, Wuhan Institute of Technology, Wuhan, China; ^2^ Department of Food Science and Chemical Engineering, Hubei University of Arts and Science, Xiangyang, China

**Keywords:** metal–organic frameworks (MOFs), electrochemical sensors, composites, sensitivity, stability

## Abstract

Developing efficient catalysts is vital for the application of electrochemical sensors. Metal–organic frameworks (MOFs), with high porosity, large specific surface area, good conductivity, and biocompatibility, have been widely used in catalysis, adsorption, separation, and energy storage applications. In this invited review, the recent advances of a novel MOF-based catalysts in electrochemical sensors are summarized. Based on the structure–activity–performance relationship of MOF-based catalysts, their mechanism as electrochemical sensor, including metal cations, synthetic ligands, and structure, are introduced. Then, the MOF-based composites are successively divided into metal-based, carbon-based, and other MOF-based composites. Furthermore, their application in environmental monitoring, food safety control, and clinical diagnosis is discussed. The perspective and challenges for advanced MOF-based composites are proposed at the end of this contribution.

## Introduction

With the development of science and technology, the demand for substance detection is becoming more selective (Sharma and Mutharasan, 2013). Sensors can effectively respond to an electrical, optical, or other signal in the presence of the analyte, and then, it will convert the physical parameters to complete the detection of the substance (Justino et al., 2015). The sensor system includes an identification element, a sensor, and a detector, which can be divided into several sensors (Chiu et al., 2017; Gogotsi et al., 2017; Zhou et al., 2017; Li S. et al., 2018; Shankar et al., 2018; Liu et al., 2021; Lin et al., 2022; Lu et al., 2022; Zamzami et al., 2022). Among them, electrochemical sensors, with simplicity, strong selectivity, and high sensitivity, have attracted wide attention (Yang S et al., 2021). Recently, nanomaterials with the advantages of high specific surface area, excellent catalytic performance, conductivity, and biocompatibility, can help the electrochemical sensors amplify signals and improve the sensitivity of the sensors as well as reduce the detection range (Yang Q et al., 2017). An efficient electrochemical sensor requires two requirements: high specificity of the signal tag and an electrode with superior sensitivity and stability (Yang ZH et al., 2017; Yang et al., 2022).

Metal–organic frameworks (MOFs) are known as coordination polymer networks or porous coordination polymers (Rosi, 2003; Zhang and Lin, 2014). The structure of different target molecules can be designed by selecting metal coordination nodes and organic junctions. Importantly, a metal-center (e.g., electrical, catalytic, or magnetic), an organic ligand (e.g., luminescent, fluorescent, or chiral), or a combination of both may produce a universal framework function that exceeds the accessible porosity (Liu et al., 2018). However, the slow mass transfer, low conductivity, and instable structure of MOF as catalysts limit their practical application (Ma and Zhu, 2020). MOF-based composites present higher surface area and richer active sites and exhibit highly ordered pore-like arrangement, which can expose active sites to a greater extent and make them have higher catalytic activity (Qin et al., 2018; Ma et al., 2020a; Ma et al., 2020b).

Recently, different effective strategies have been proposed to modify MOFs for improved electrocatalytic behavior, mechanical properties, and stability. Generally, metal nanoparticles, with the advantages of superior conductivity and high surface area, can be used to increase the electron transfer rate (Samadi-Maybodi et al., 2015; Da Silva et al., 2016). Thus, MOF–metal nanocomposites present versatility, high stability, and dispersibility. As a result, MOF composite with metal particles is an effective route to design the superior electrochemical sensors (Meng et al., 2018). MOFs composited with noble metals, transition metals, and two different metal cations are regarded as effective strategies to prepare sensing materials with higher stability and catalytic efficiency (Nan et al., 2020; Yang et al., 2020). Furthermore, MOFs show low electronic conductivity, electrical reactivity, and stability in aqueous media, which limit their applications in electrochemical sensors. Assembling MOFs with conductive materials, such as graphene, carbon nanotubes, carbon blocks, and carbon nanofibers, is an effective strategy (Wang et al., 2017; Lai et al., 2019; Li L et al., 2019; Zhou Y. et al., 2020). In addition, doping graphene with heteroatom can further improve the catalytic activity of MOF-based carbon materials (Deng et al., 2020a). Similar to carbon nanomaterials, conductive polymers present excellent electrical conductivity, low cost, and ease of polymerization that are ideal materials to overcome poor electrical conductivity of MOFs (Liu et al., 2020). The introduced nonnatural polymers, such as new functional groups, can significantly improve the structure and properties of MOFs. Moreover, by integrating heme into MOFs, the dimerization and oxidative self-destruction of heme are improved, contributing to their optimum detection performance and stability (Zhang et al., 2015).

In this review, advances of MOF-based catalysts in electrochemical sensors are comprehensively summarized. Based on the structure–activity–performance relationship of MOF-based catalysts, we introduce the mechanism of MOF-based catalysts as electrochemical sensors, including metal cations, synthetic ligands, and structure. Then, the MOF-based composites are successively divided into metal-based, carbon-based, and other MOF-based composites. Furthermore, their application in environmental monitoring, food safety control, and clinical diagnosis is discussed. The perspective and challenges for advanced MOF-based composites are proposed at the end of this contribution.

## Mechanism of Metal–Organic Frameworks-Based Catalysts in Electrochemical Sensor

MOFs, with high porosity, biocompatibility, and superior specific surface area, have been widely applied in catalysis, adsorption, separation, and energy storage (Jiawen et al., 2019). The mechanism of MOFs in electrochemical sensors is as follows: 1) Signal amplification: loading different functional materials and signal molecules on MOFs is beneficial to the electrochemical detection (Yi et al., 2016; Liu et al., 2020). 2) Catalysts and signal probes: MOFs present periodic porous structure by coordination of metal cations and organic ligands, which can induce rich catalytic activity and redox activity centers, attributing to superior electrochemical properties (Liu et al., 2018). 3) Size selection: the macroporous structure of MOFs is helpful to easily introduce the guest materials and perform the size selection of substance molecules (Zhu and Xu, 2014; Zhang et al., 2019). 4) MOFs can generate interaction forces with analytes (including Van der Waals force, covalent bond, and p–p interaction), resulting in the improved selectivity for electrochemical detection (Carrasco, 2018).

In this review, the mechanism of MOFs in catalysts and signal probes, including the effects of catalytic active centers and redox active centers on electrochemical sensors, is mainly discussed (Liu et al., 2018). MOF materials with metal cations and ligands can provide the desired active sites, which deserve high catalytic activity for various detection molecules. In the electrochemical sensors, metal ions can be used as charge carriers. The interaction between the sensing material and target analyte is beneficial to enhance its selectivity at room temperature (Li et al., 2021). Furthermore, the active metal ions in MOF-based nanomaterials can be used as the catalysts, which can improve the activity of the oxidation–reduction reaction, resulting in the amplified electrochemical signals and the improved sensitivity (Liu et al., 2018). Furthermore, the metal cations in MOFs also act as coordination centers to form an infinite crystal network. For example, common active metal nodes, such as Co, Cu, Zn, and Cr, and their redox activity can enhance the catalytic ability of MOFs (Liu et al., 2020). In addition, organic ligands with redox activity are also attributed to the catalytic active sites of MOFs (Yang et al., 2022). In general, the organic ligands for MOFs can be divided into chemical ligands and biological ligands (Smaldone et al., 2010). For instance, porphyrin, heme, and amino acids are common organic ligands, which present affinity to metal cations and combine well with them.

The superior structure of MOFs also plays an important effect on the boosted electrochemical activity. Their flexible and highly porous structure can be helpful to the easy diffusion of analyte molecules, facilitating the interaction between the host and analyte (Liao et al., 2018; Yang et al., 2022). Furthermore, MOFs, with a porous structure and superior specific surface area, act as a good carrier to form composite materials, resulting in the improved electrochemical activity (Kitagawa et al., 2004; Cui et al., 2013; Wang C et al., 2018; Li D et al., 2019; Wang Y et al., 2020). Therefore, regulating the structure of MOFs is an effective route to adjust its composition and structure, leading to larger surface area, higher porosity, and better electrochemical activity (He et al., 2021).

## Metal–Organic Framework-Based Composites

MOFs, with the merits of diverse chemical combinations, rich metal active sites, and adjustable structure, have attracted wide attention (Li et al., 2020). However, the slow mass transfer, low conductivity, and instable structure of MOFs as catalysts limit their practical application (Ma and Zhu, 2020). Various strategies are adopted to improve the electrocatalytic behavior, mechanical properties, and stability of MOFs. Designing MOF–metal, MOF–carbon, and other MOF-based nanocomposites is an effective strategy to induce MOFs with high porosity and ordered crystal pores (Bradshaw et al., 2012; Moon et al., 2013; Qiang et al., 2013; Falcaro et al., 2014; Li et al., 2016).

### Metal–Organic Framework–Metal Nanocomposites

Due to the limited pore size of MOFs, the size of synthesized particles will be confined to nanoscale. Metal nanoparticles, with the advantages of superior conductivity and high surface area, can be used to increase the electron transfer rate (Ghaffari et al., 2015; Da Silva et al., 2016). Generally, MOF–metal nanocomposites present versatility, high stability, and dispersibility. Therefore, MOF composite with metal particles is an effective route to design the superior electrochemical sensors (Meng et al., 2018).

**FIGURE 1:**
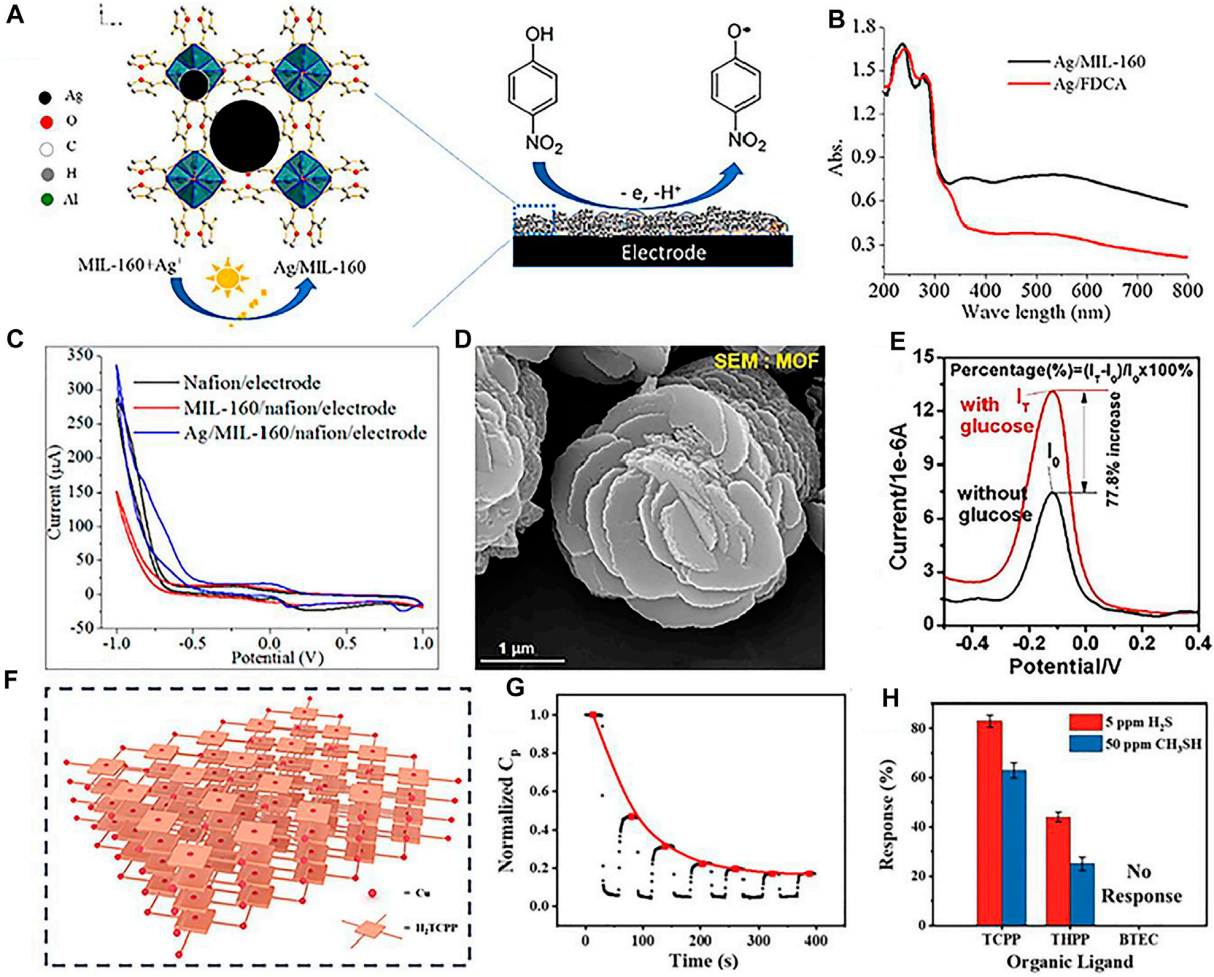
**(A)** Synthetic process of Ag/MIL-160; **(B)** UV–vis diffuse reflectance spectra of prepared composites; **(C)** CV curves of different electrodes at a scan rate of 50 mV s^−1^ in an electrolyte with *p*-NP. Reproduced with permission (Liu et al., 2019). Copyright 2019, ACS. Scanning electron microscope (SEM) image **(D)** and DPV responses without and with 4 mM glucose **(E)** of Cu(II)-anchored MOFs. Reproduced with permission (Zhang et al., 2020). Copyright 2020, Elsevier. Scheme **(F)**, normalized capacitance response **(G)** of the Cu-TCPP IC-MOF sensor and **(H)** Cu-X IC-MOF sensors to 5 ppm H_2_S and 50 ppm CH_3_SH. Reproduced with permission (Li et al., 2021). Copyright 2020, Wiley.

The size and morphology of noble metal nanoparticles can reduce the overpotential of oxidation and reduction, which can effectively regulate the electrocatalytic properties (Azad and Ganesan, 2010; Gupta and Ganesan, 2015; Sonkar and Ganesan, 2015). Recently, grafting noble metal nanoparticles on MOFs is widely applied as new electrode materials for various electrochemical and biochemical sensors (Turner et al., 2008; Sabo et al., 2007; Zhu et al., 2017; Mosleh et al., 2017). For instance, a porous rhombic dodecahedron structure of Ag@zeolitic imidazolate framework-67 is synthesized, which presents a strong electrocatalytic activity and low detection limit toward H_2_O_2_ reduction (Dong et al., 2019). Interestingly, combined with photocatalytic technology and electrochemical sensing, a novel synthetic method of Ag/MIL-160 hybrid is developed to detect p-nitrophenol (Figure 1A) (Liu Q. et al., 2019). The generated charge carrier in the Ag/MIL-160 organic molecule initiates its photocatalytic functionality (Figure 1B), the high sensitivity for pollutant reduction is dictated by the photocatalytic activity of Ag nanoparticles, and selective electron migration on the electrode interface (Figure 1C). In another example, Ag/MIL-101 composite-modified GCE is reported to be useful for monitoring tryptophan (Peng et al., 2016). The existed p–p accumulation between the ligands of MOF and tryptophan increases the diffusion of analyte molecules. Furthermore, the electromagnetic field generated by the noble metal nanoparticles promotes the accumulation of tryptophan molecules on the surface of MIL-101 (Yang J et al., 2015).

Transition metals, such as Cu, Fe, Co, Ni, Zn, and Mg, present the merits of low price and high efficiency; they have been widely introduced to composites with MOF (Li et al., 2019; Hosseini et al., 2016). For instance, Cu(II)-anchored MOFs are constructed as signal probes (Figure 1D) (Zhang et al., 2020). This prepared composite presents an excellent glucose oxidation activity and amplified electrochemical signal, which can be ascribed to the oxidation of glucose by the generated Cu(III) from the oxidation of Cu(II) (Figure 1E). Furthermore, ionic conductive metal–organic framework sensor arrays act as charge carriers, which can directly and selectively interact with analytes (Lu et al., 2020). For example, a series of IC-MOF sensor arrays are constructed by modulating various metal nodes (Cu, Co, Ni, Zn, and Mg) and organic ligands (H_2_TCPP, H_2_THPP, and H_4_BTEC) (Figure 1F) (Li et al., 2021). Due to the strong interaction between H_2_S and Cu^2+^, the synthesized material can generate CuS during the detection process, resulting in an irreversible reaction, which can be adopted to detect volatile sulfide (Figures 1G,H).

The bonding between two different metal cations can increase electrical conductivity and improve the electrocatalytic efficiency, which is ascribed to different oxidation potentials and associated electron configurations (Yang et al., 2018). Therefore, a unique synergistic effect between two different metal elements is helpful in obtaining higher stability and efficiency (Wen et al., 2015; Jiang et al., 2018). Recently, bimetallic nanoparticles, including Fe, Co, Ni, or Cu, with a cooperative effect have been developed to optimize the performance of MOFs (Tang et al., 2016; Wang Z. et al., 2018). For instance, Au@Cu MOF nanomaterials with unique structures can effectively increase the number of binding sites on the polymer network, obtaining a more sensitive electrochemical sensor (Hatamluyi et al., 2020). The advanced core–shell heterostructure is introduced to fabricate composites by encapsulating metal oxides or metal nanoparticles as a core and MOFs as a shell (Paolo et al., 2016; Wengert et al., 2017; Yang Q. et al., 2017). The metal oxides or metal nanoparticle cores (e.g., magnetic, electrical, and catalytic properties, etc.) act as a catalyst, and MOFs shells (e.g., multiple coordination sites, ordered crystalline pores, structural adaptability advantages, and flexibility, etc.) act as a recognition agent for analog molecular sieves, may be combined. These advanced structures can greatly improve their anti-aggregation stability and avoid undesirable dissolution or corrosion in the photocatalytic process, resulting in boosted catalytic and adsorption properties (Liu and Tang, 2013; Kempahanumakkagari et al., 2018). For instance, Fe-MOF@Fe_3_O_4_@C core–shell nanostructured composite is composed of iron-based MOF and mesoporous Fe_3_O_4_@C (Zhang et al., 2017). Specific aptamer metal ions (e.g., Pb^2+^ and As^3+^) are attached to the constructed nanocomposites by supramolecular stacking and hydrogen bond interactions, exhibiting good anti-interference characteristics and detection of Pb^2+^ and As^3+^ ions in spiked river water.

### Metal–Organic Framework–Carbon Nanomaterial Composites

The weak electronic conductivity, electrical reactivity, and low stability in aqueous media of MOFs limit their applications in electrochemical sensors. To overcome these technical shortcomings, they can be assembled with conductive materials, such as graphene, carbon nanotubes, carbon blocks, and carbon nanofibers, which are introduced to be assembled with MOFs (Wang et al., 2017; Lai et al., 2019; Li Y et al., 2019; Zhou et al., 2020). Among which, graphene oxide/reduced graphene oxides/carbon nanotubes as unique conductive additives can improve the electrical conductivity and mechanical strength of MOFs (Zhang et al., 2014; Liu et al., 2016).

**FIGURE 2:**
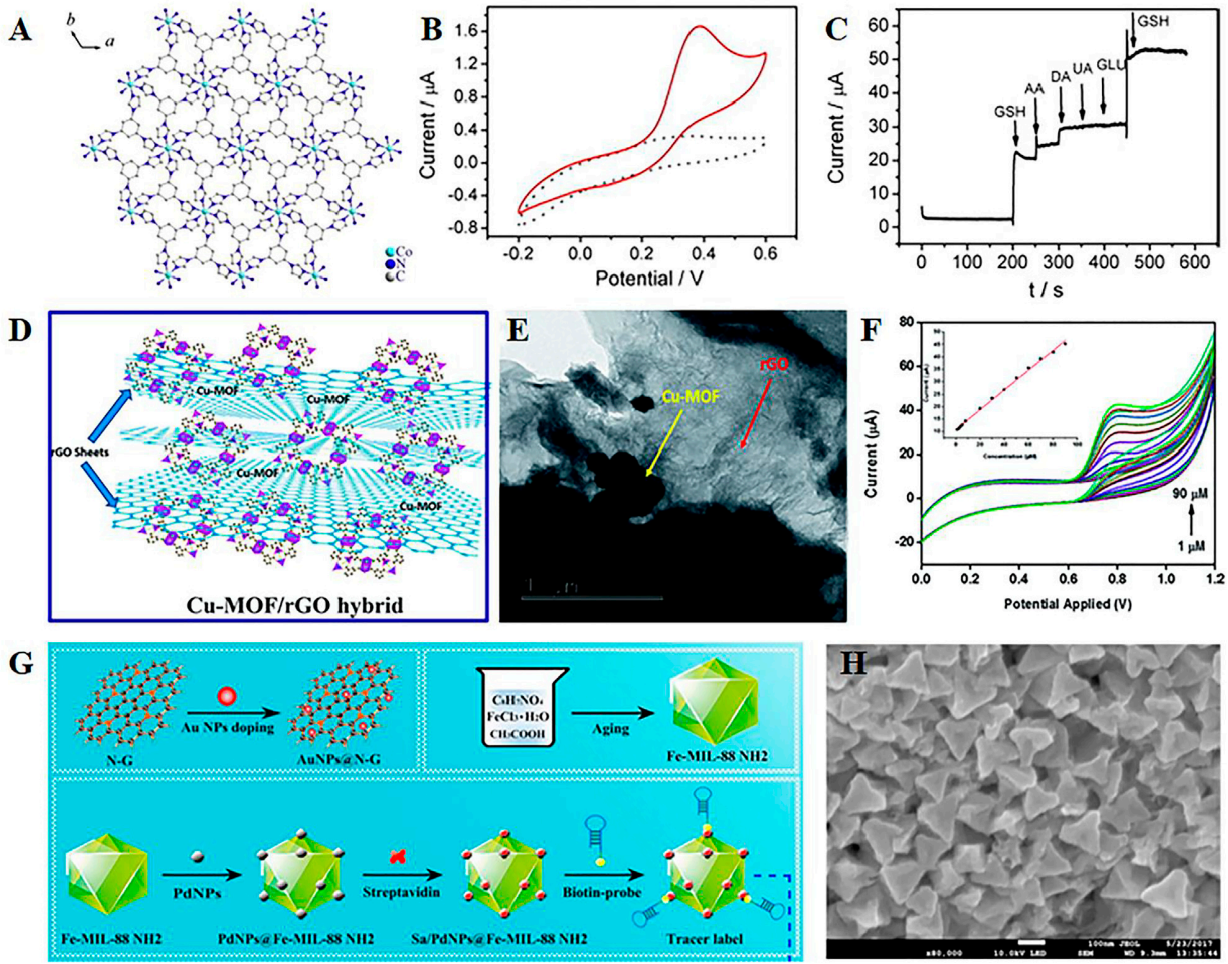
**(A)** Illustration of the structure of metal–organic coordination polymers with 1,3,5-tris (1-imidazolyl) benzene and transition element Co^2+^ (Co-MOCP), CVs **(B)**, and interference test **(C)** of the Co-MOCP/carbon paste electrode in the different solution. Reproduced with permission (Yuan et al., 2014). Copyright 2014, Elsevier. Schematic structure **(D)**, transmission electron microscope (TEM) images **(E)**, and CV curves at different nitrite concentrations **(F)** of the Cu-MOF/rGO hybrid. Reproduced with permission (Saraf et al., 2016). Copyright 2016, RSC. **(G)** Synthetic route of different composites; **(H)** field emission scanning electron microscope image of PdNPs@Fe-MOFs. Reproduced with permission (Li et al., 2018). Copyright 2018, Elsevier.

The simplest way to improve the conductivity of MOF materials is to mix them with highly conductive carbon paste electrodes (Yang et al., 2014; Wang et al., 2013). The pore structure of carbon paste electrode-modified MOFs composite can allow the analyte to be pre-concentrated from the bulk solution onto the electrode surface, which helps to improve the selectivity of the analyte (Doménech et al., 2007). For instance, Co-based metal–organic coordination polymer-modified carbon paste electrodes are developed to analyze the electrocatalytic performance of redox glutathione (Figure 2A) (Yuan et al., 2014). This constructed composite exhibits excellent electrocatalytic oxidation–reduction and high selectivity of glutathione (Figures 2B,C). Furthermore, carbon paste electrodes modified with MOFs can be used for the electrocatalytic oxidation and detection of nitrite (Zhou et al., 2014). The modified material demonstrates improved sensitivity and selectivity. However, high background currents and continuous use of the electrode material will lack stability and reproducibility. Carbon nanotubes, with the advantages of the high aspect ratio, large specific surface area, and good mechanical properties and electrical properties, are widely used as electrode materials (Chen and Dai, 2013). Single-walled carbon nanotubes (SWCNTs) are covalently functionalized with benzoic acid and transition metal ions, which can form a 3D porous inorganic–organic hybrid framework, resulting in good electrochemical performance and reproducibility. Therefore, SWCNT–MOF composite is an effective electrochemical sensor material for organophosphorus pesticides.

Similarly, graphene is a well-known advanced two-dimensional nanomaterial with advantages of large specific surface area and ultrafast carrier mobility (Chen et al., 2013). Therefore, MOF/graphene (or graphene oxide) composites have been developed in various electrochemical sensing applications. A copper-based MOF is proposed by combining with graphene for electrochemical sensing of H_2_O_2_ and ascorbic acid (Yang T et al., 2015). Due to the hydrogen bond between Cu-MOF and graphene, p-p stacking, and Cu-O coordination, the synthesized nanocomposites exhibit high stability and good anti-interference properties detect H_2_O_2_ and ascorbic acid in various carbohydrates. Furthermore, rGO can be introduced to the composite with MOF for detection of nitrite 1 (Figure 2D) (Saraf et al., 2016). rGO can greatly improve the conductivity of MOF in the composite. The positive synergistic effects exist between Cu-MOF crystals and rGO nanosheets (Figure 2E), and these can be attributed to the improved electrocatalytic performance of the prepared electrochemical sensor electrode (Figure 2F).

In general, nitrogen-doped graphene can present more defect sites and lower aggregation of graphene sheets, which is beneficial to increase the biocompatibility of graphene sheets and functionalize easily with noble metal nanoparticles (Chen et al., 2016). A signal amplification strategy is developed to construct AuNPs-functionalized nitrogen-doped graphene as capture probes, and PdNPs@Fe-MOFs as nanocarriers (Figures 2G,H) (Li et al., 2018). This assembled structure can initiate the next reaction process, which induces numerous tracer indicators anchored onto the sensing interfaces, contributing to the superior specificity and recovery in spiked serum samples. In addition, black phosphorus can bind with antibodies and enhance electron transfer; thus, the black phosphorus electrochemical sensor based on magnetic covalent organic frameworks is developed to detect prostate-specific antigens, which can be widely applied in detecting biomarkers of cancer (Pandey et al., 2021).

### Other Metal–Organic Framework-Based Composites

**FIGURE 3:**
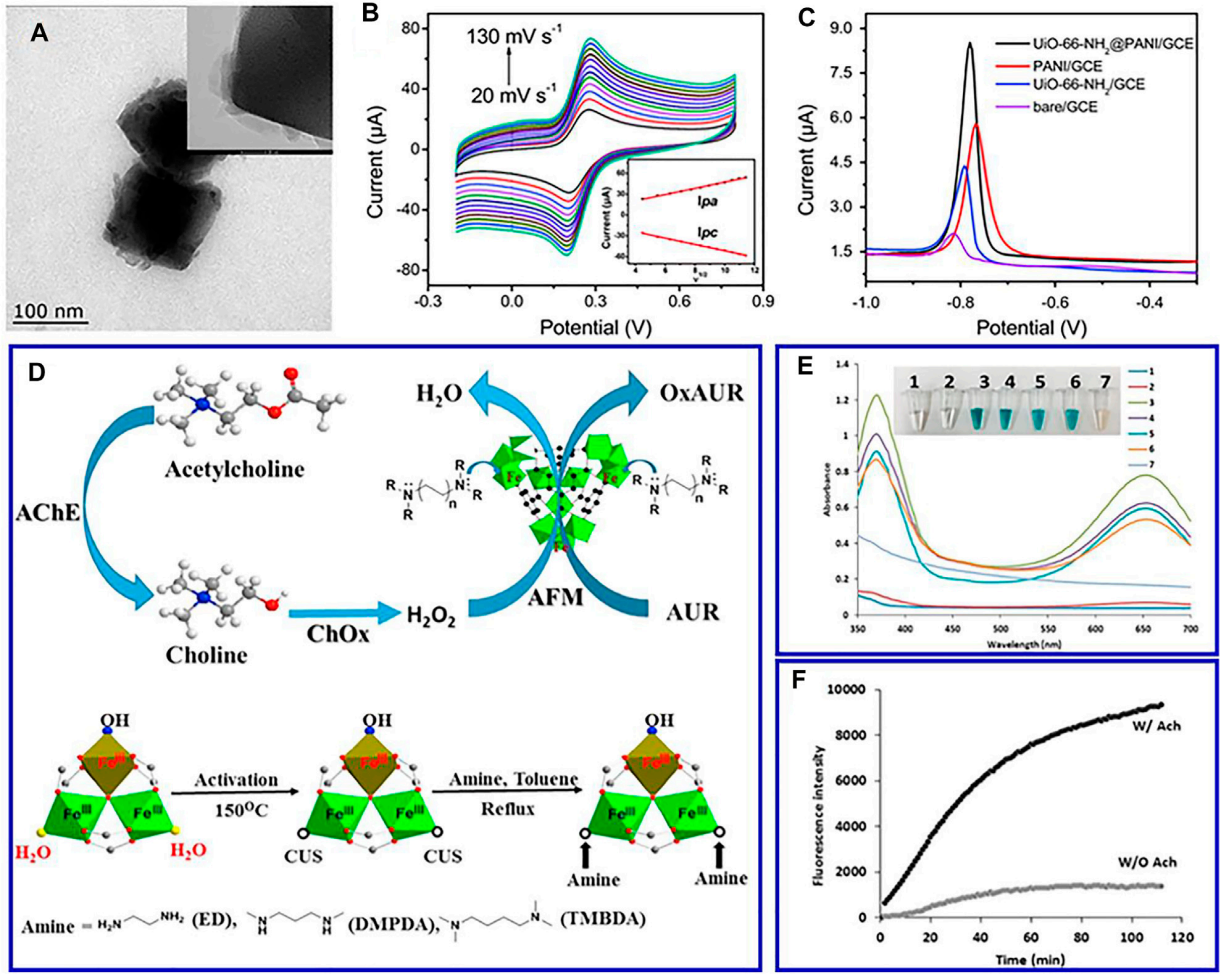
TEM images **(A)**, CV in 0.1 mol L^−1^ KCl solution with 1 mmol L^−1^ Fe(CN)_6_^3−/4−^ at different scan rates **(B)**, and differential pulse voltammograms of 100 μg L^−1^ Cd(ii) **(C)** of prepared UiO-66-NH_2_@PANI. Reproduced with permission (Wang et al., 2017). Copyright 2017, RSC. **(D)** Schematic representation of the route and coordinatively unsaturated grafted MIL-100(Fe); **(E)** comparison of the peroxidase-mimic activity of different samples; **(F)** time-dependent fluorescence intensities. Reproduced with permission (Valekar et al., 2018). Copyright 2018, Elsevier.

Similar to carbon nanomaterials, conductive polymers present excellent electrical conductivity, low cost, and ease of polymerization that are ideal materials to overcome poor electrical conductivity of MOFs (Liu et al., 2020; Deng et al., 2020b). For instance, MOF–polyaniline composite (UiO-66-NH_2_@PANI) was synthesized by polymerizing a conductive PANI in the presence of pre-synthesized UiO-66-NH_2_ (Wang et al., 2017). The composite exhibited excellent electrochemical redox performance of Cd^2+^ ions, which is related to the synergistic effect between UiO-66-NH_2_ and PANI (Figures 3A–C). Furthermore, the large surface area and the existing chelating groups in MOF increase the number of conduction paths and increase the electron transfer rate between the solution and the composite electrode surface. The introduced nonnatural polymers, such as new functional groups, can significantly improve the structure and properties of MOFs. MIL-53(Fe), a flexible material consisting of iron oxides and phthalate, is functionalized by polymethyl methacrylate and can be used to make electrochemical sensor materials for detecting melamine in milk samples (Yang Z et al., 2021). In addition, MOFs have the merits of abundant pores, large surface area, and good biocompatibility, which can effectively prevent the aggregation and leakage of enzymes and improve the biological activity and stability of enzymes (Liu et al., 2020). Therefore, MOFs are usually used to immobilize enzyme and other biomacromolecules.

Heme is a famous natural metalloporphyrin, which acts as the active center of hemoglobin. Due to the reversible conversion of Fe(III)/Fe(II), heme presents significant peroxidase-like catalytic activity (Reuillard et al., 2017). However, its catalytic life is limited due to dimerization and oxidative self-destruction in the aqueous medium (Li et al., 2018). Anchoring hemin on a suitable carrier material is an effective strategy to remedy these shortcomings (Wang et al., 2016). MOFs with a regular porous structure are ideal candidates for hemin fixation (Zhang et al., 2015). By integrating heme into MOFs, the dimerization and oxidative self-destruction of heme are improved, contributing to the optimum catalytic performance and chemical stability of MOFs. For instance, a novel amine-grafted MOFs is designed as a promising alternative to peroxidase enzyme (Figure 3D) (Valekar et al., 2018). The synergetic effect of the enhanced negative potential and tuned molecular size of the grafted diamine are attributed to the improved fluorescent assay of choline and acetylcholine, which effectively detect choline and acetylcholine levels in real samples of milk and serum (Figures 3E,F).

## Application of Metal–Organic Framework-Based Catalysts in Electrochemical Sensors

### Environmental Monitoring

With rapid urbanization and industrialization, the ecological environment is suffering from serious damage (He et al., 2021). Developing satisfactory electrochemical sensors is an effective route to detect harmful chemicals, especially the detection of toxic gases with low concentrations and heavy metal ions in water (Dorda et al., 2003; Aslam et al., 2014; Zhang et al., 2017). MOF-based composites can be used to transform harmful chemicals in air and water into electrochemistry as fine-sensing materials. However, the formation of MOF inorganic clusters is highly dependent on the geometry, length, and connectivity of building organic linkers (Bai et al., 2016; Cho et al., 2019). Therefore, expanding the diversity and properties of MOFs is critical to their applications in environmental monitoring.

**FIGURE 4:**
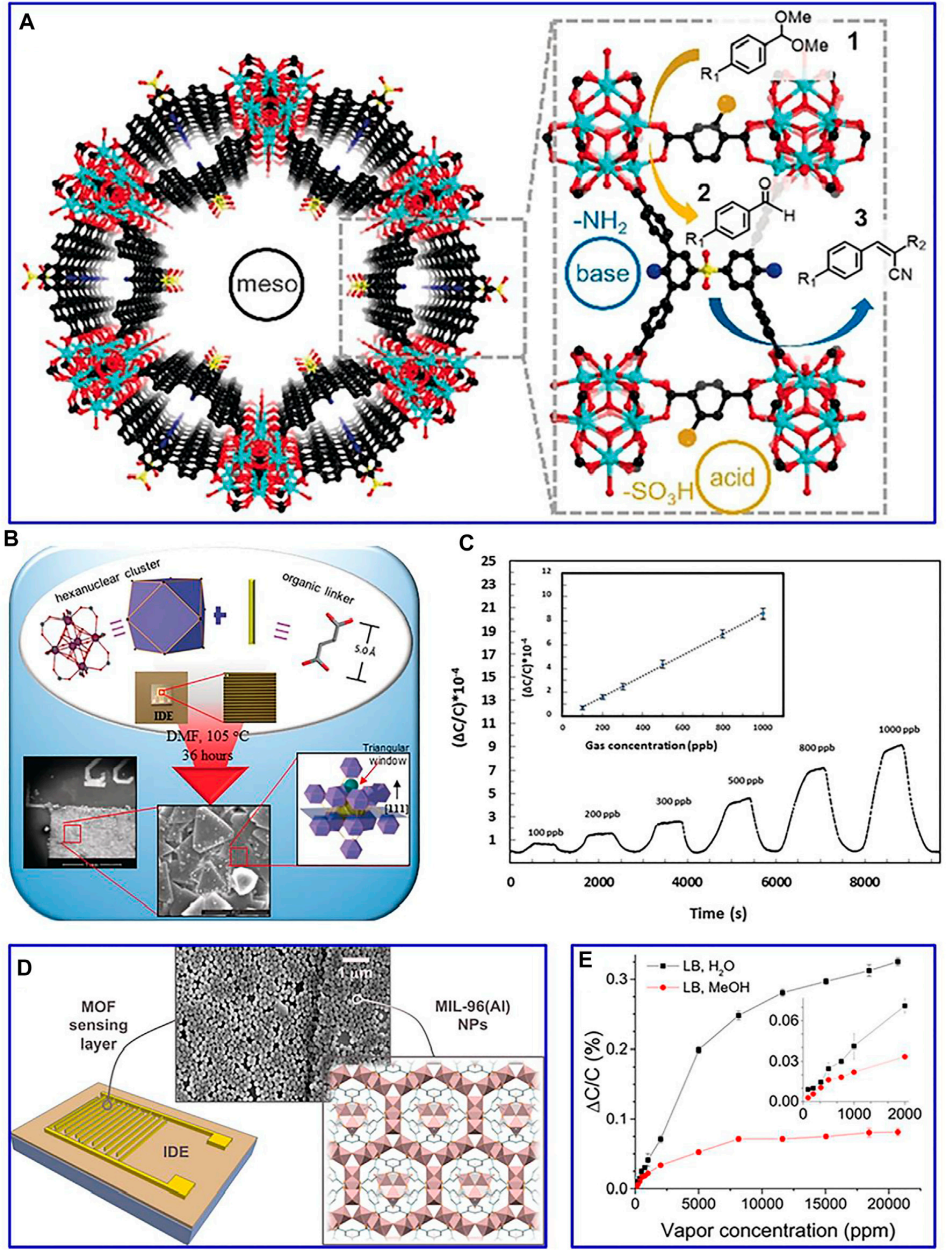
**(A)** One-pot tandem reaction of benzaldehyde dimethylacetal and malononitrile by bifunctional mesoporous catalyst. Reproduced with permission (Feng et al., 2019). Copyright 2019, Wiley. **(B)** Schematic of the prepared approach of the fumarate-based fcu-MOF; **(C)** detection of H_2_S with concentrations of 1–100 ppm. Reproduced with permission (Yassine et al., 2016). Copyright 2016, Wiley. **(D)** Illustration of the structure of the used IDEs showing the MOF LB film characterization by SEM; **(E)** normalized capacitive response of IDEs to water and methanol. Reproduced with permission (Andrés et al., 2020). Copyright 2020, ACS.

For instance, a layered porous Cu–benzene-1,3,5-tricarboxylic acid MOF is constructed for the glyphosate detection (Cao et al., 2019). The response current of the synthetic material is significantly increased, which can be ascribed to the strong affinity between chelate groups on the glyphosate with Cu^2+^. In another example, gold-modified MoS_2_/rGO and AuPd@Fe-MOFs are constructed as an electrochemical adapter sensor for detecting Pb^2+^ (Wang Z et al., 2020). The combination of catalytic chain and base complementary is contributed to the improved detection performance. Interestingly, the hinge-like organic ligand is obtained by the desymmetrization strategy (Feng et al., 2019). MOF materials are modified at the molecular level, which can realize the coexistence of acidic sites and alkaline sites in the material. This synthetic mesoporous composite with a functional structure is beneficial to the transformation of cascade catalytic, achieving high activity of a two-step efficient series catalytic reaction (Figure 4A).

MOF-based composites with the merits of excellent gas adsorption/separation capability can be adopted as electrically transduced gas sensors (Yao et al., 2021). Integrating MOFs onto capacitive sensors based on interdigitated electrode chips is an effective route to improve their detection performance. For example, an *in situ* growth strategy is adopted to synthesize fumarate-based fcu-MOF thin film on an interdigitated electrode (Figure 4B). This constructed sensor presents a remarkable detection sensitivity (down to 100 ppb) and lower detection limit (around 5 ppb) for H_2_S (Figure 4C). Furthermore, the Langmuir–Blodgett method is used to deposit MIL-96(Al) MOF thin films on the interdigitated electrode chips (Figure 4D) (Andrés et al., 2020). These prepared films achieve superior selective and short response/recovery for water and methanol (Figure 4E), which can be also extended to the detection of methanol, toluene, and chloroform, etc.

### Food Safety Control

Electrochemical (biological) sensors are one of the most sensitive, simple, and selective chemical sensors, which have been widely used in rapid and reliable food safety control (Otles and Yalcin, 2012). MOFs, with the merits of uniform structures, ultrahigh porosity, and tunable composition, act as the promising sensor for food safety control. Nevertheless, the reuse and long-term storage of electrochemical sensor materials fabricated from MOFs in complex sample matrices remain a challenge. Inexpensive microbial sensors are designed for single use to avoid degradation of biosensor elements in complex matrices (Semih Otles, 2012).

**FIGURE 5:**
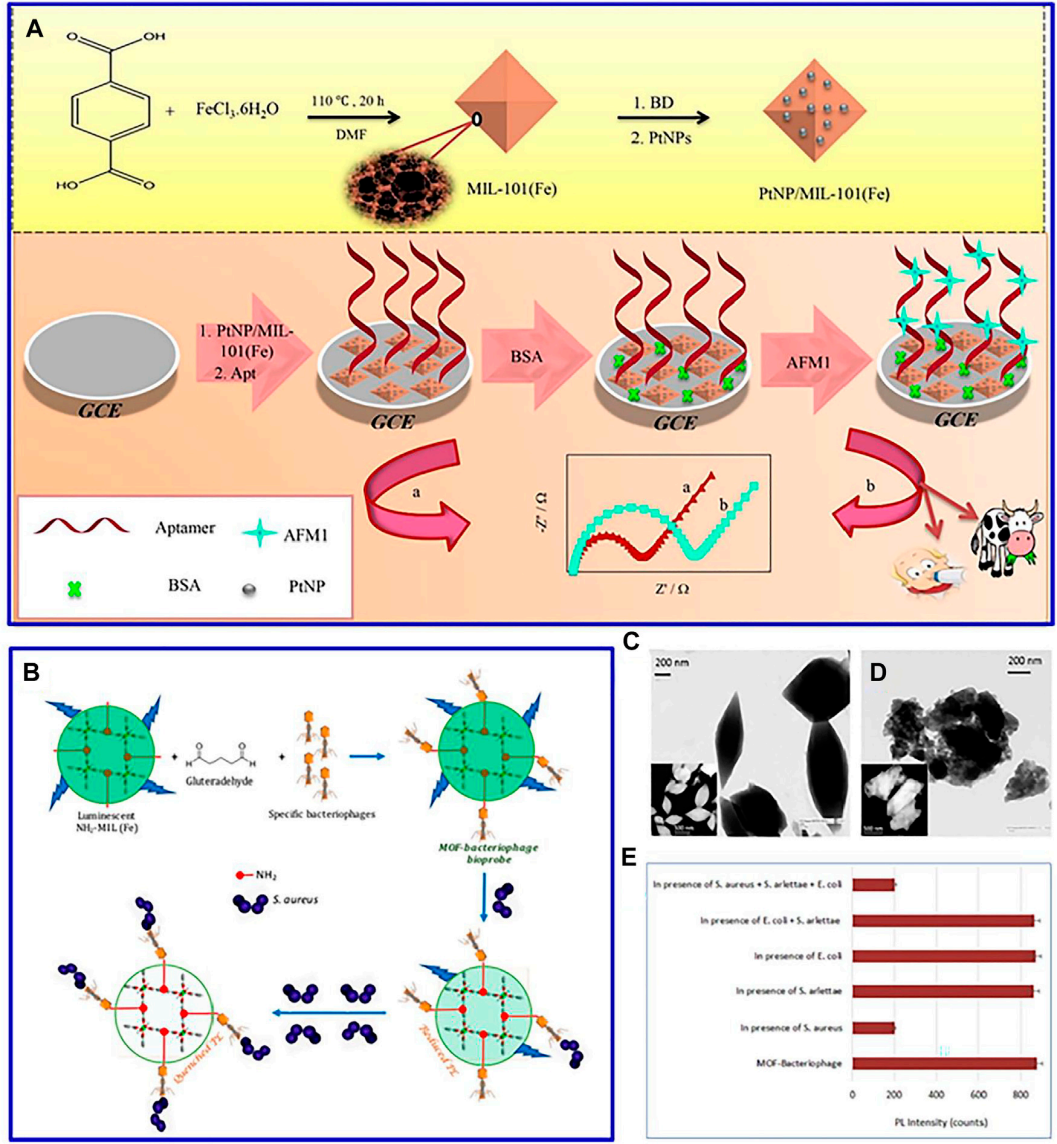
**(A)** Schematic diagram for the preparation schematic diagram and electrochemical test of PtNP/MIL-101(Fe). Reproduced with permission (Jahangiri-Dehaghani et al., 2020). Copyright 2020, Elsevier. **(B)** Schematic of NH_2_-MIL53(Fe)-bacteriophage biosensor; TEM image of **(C)** NH_2_-MIL-53 MOF and **(D)** bacteriophage-loaded NH_2_-MIL-53 MOF; **(E)** specificity of the proposed biosensor. Reproduced with permission (Bhardwaj et al., 2017). Copyright 2017, ACS.

In a recent study, Pt nanoparticles were decorated on a glassy carbon electrodes-modified Fe-based MOFs, and this designed MOF-based composites acted as a sensitive label-free electrochemical aptasensor to detect aflatoxin M1 (Figure 5A) (Jahangiri-Dehaghani et al., 2020). The fabricated aptasensor was successfully applied to measure AFM1 concentration in powder and pasteurized milk samples. Furthermore, molecularly imprinted mMOFs was synthesized by layer–layer modification to detect turtotomycin (Li et al., 2021). The magnetic pole in molecularly imprinted mMOFs is beneficial to form an electrochemical sensing interface, and its imprinted cavity can serve as electronic channels for probes for label-free detection of over-the-counter drugs.

In general, when food with packaging is stored for a long time, foodborne pathogen infection will occur, resulting in many health-related problems (Jahangiri-Dehaghani et al., 2020). This MOF-based luminescent sensor can be adopted to detect these foodborne pathogens. For instance, a fluorescent MOF [NH_2_-MIL-53(Fe)] with a target-specific bacteriophage was synthesized for the detection of *Staphylococcus aureus* (Figure 5B) (Bhardwaj et al., 2017). The advanced structure of MOF can offer a precise control on particle size distribution (Figures 5C,D), which is helpful to construct a better structural compatibility with bacteriophages, contributing to the high stability, specificity, reusability, and wider linear range of the bacteriophage sensor (Figure 5E). In addition, CeO_2_/CuO_x_@MC nanocomposite is introduced as a carrier to detect the microtobramycin in milk (Cheng et al., 2021). The combination of different materials presents a strong biological affinity for the adaptor chain, contributing to a wide linear range and low detection limit of tobramycin.

### Clinical Diagnosis

MOF materials, with the advantages of selective composition, adjustable pore size, and large surface area are widely used as electrochemical sensor materials in biomedical fields, including cancer diagnosis (e.g., cancer markers, microRNA, and live cancer cells) and glucose detection (Chen et al., 2013; Qin et al., 2016; Xie et al., 2018; Petrosillo et al., 2020). Nevertheless, it is difficult to construct the nanoparticles and active biomolecules in the same MOF-based structure. Recently, many strategies, such as functional MOFs and combination with bionic enzyme, as well as utilization of biosensing and molecular recognition technology, have been developed to improve the photoelectric and catalytic properties of MOF materials (Liao et al., 2017; Shen et al., 2018).

**FIGURE 6:**
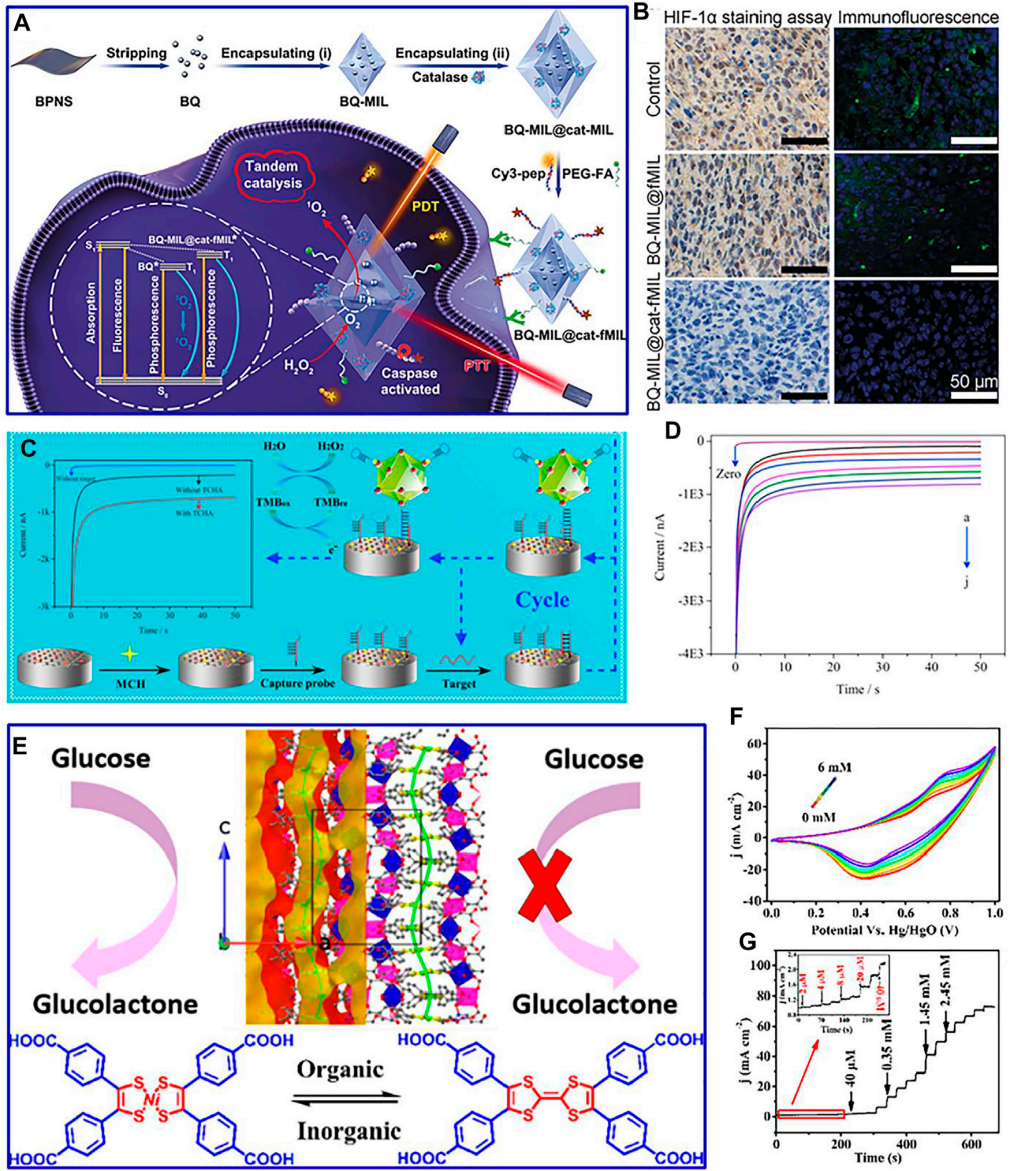
**(A)** Assemble process of synthetic material and its mechanism against hypoxic tumor cells; **(B)** immunohistochemistry and immunofluorescence staining of HIF-1α in tumor slices of different composites. Reproduced with permission (Liu et al., 2019). Copyright 2019, Wiley. **(C)** Fabrication process of the biosensor and the target-catalyzed hairpin assembly for target recycle; amperometric curves **(D)** of the proposed biosensor incubated with different concentrations of miR-122 containing 1 mM 3,3′,5,5′-tetramethylbenzidine and 20 μM H_2_O_2_. Reproduced with permission (Li et al., 2018). Copyright 2018, Elsevier. **(E)** Structures of the constructed material; CV curves **(F)** and amperometric response **(G)** of the constructed material with varied glucose concentrations. Reproduced with permission (Zhou et al., 2020). Copyright 2020, ACS.

For instance, a multifunctional homologous MOF hybrid material is designed with enhanced therapeutic effect on hypoxic tumor cells by the *in situ* growth method (Liu et al., 2019). Black phosphorus quantum dots and catalase were precisely assembled into the inner and outer layers of a layered MOF to form a multifunctional MOF heterostructure (Figure 6A). This advanced heterostructure converts excess H_2_O_2_ into O_2_ by catalase wrapped in its outer shell, improving the hypoxic microenvironment of tumor cells (Figure 6B). An innovative MOF-on-MOF method is adopted to construct Zn-MOF-on-Zr-MOF composite for detecting protein tyrosine kinase-7 (Nan et al., 2019). The synthetic Zn-MOF-on-Zr-MOF composite presents hierarchical cross leaves and multilayer nanosheet structures, which demonstrate excellent sensing capabilities for the detection of protein tyrosine kinase-7. This improved performance is mainly ascribed to the presence of Zr-MOF, which significantly facilitates aptamer fixation and stabilizes the formed G-tetrexes, providing a new avenue for the application of bimetallic MOFs in the early cancer diagnosis. Interestingly, multifunctional iron-based MOFs, with the advantages of superior peroxidase-like activity, are developed as a sandwich-type biosensor (Figure 6C) (Li et al., 2018). The biosensor demonstrates a low detection limit (0.003 fM) and wide detection range (0.01 fM to 10 p.m.) for detecting miR-122 in human serum (Figure 6D). This strategy can be adopted to detect drug-induced liver injury at an early stage.

Recently, commercial glucose sensors are mainly based on glucose oxidase-assisted electrooxidation. In order to avoid low reproducibility, complex immobilized enzyme process, and decreased enzyme activity of commercial glucose sensors, based on the direct electrocatalysis of electrode materials, the application of MOFs in nonenzymatic sensors has been designed and developed (Burke and Gorodetsky, 2012). Nevertheless, the existing small detection range, low sensitivity, and poor stability of MOFs in nonenzymatic glucose electrochemical sensors limit their commercial application (Al-Zoubi et al., 2020). Therefore, it is vital to develop MOF-based composites with good stability and high activity for glucose detection. For example, the Au/Cu MOFs coupled with a capture probe and a convertase form a bioconjugate (Liu et al., 2020). This modified electrode is incubated in sucrose solution, which can effectively detect glucose. Furthermore, coordinating metals with the functional tetrathiafulvalene core is an insightful route to modulate the catalytic performance of MOF-based composites. For instance, nickel bis(dithiolene-dibenzoic acid) as a redox-active linker is constructed for functional MOFs (Figure 6E) (Zhou et al., 2020). This synthetic composite presents high sensitivity, wide detection range, and low detection limit for the detection of glucose (Figures 6F,G), which is ascribed to the oxidation of glucolactone by several reversible and stable oxidation states of nickel bis(dithiolene) compounds.

## Perspective and Prospect

**FIGURE 7:**
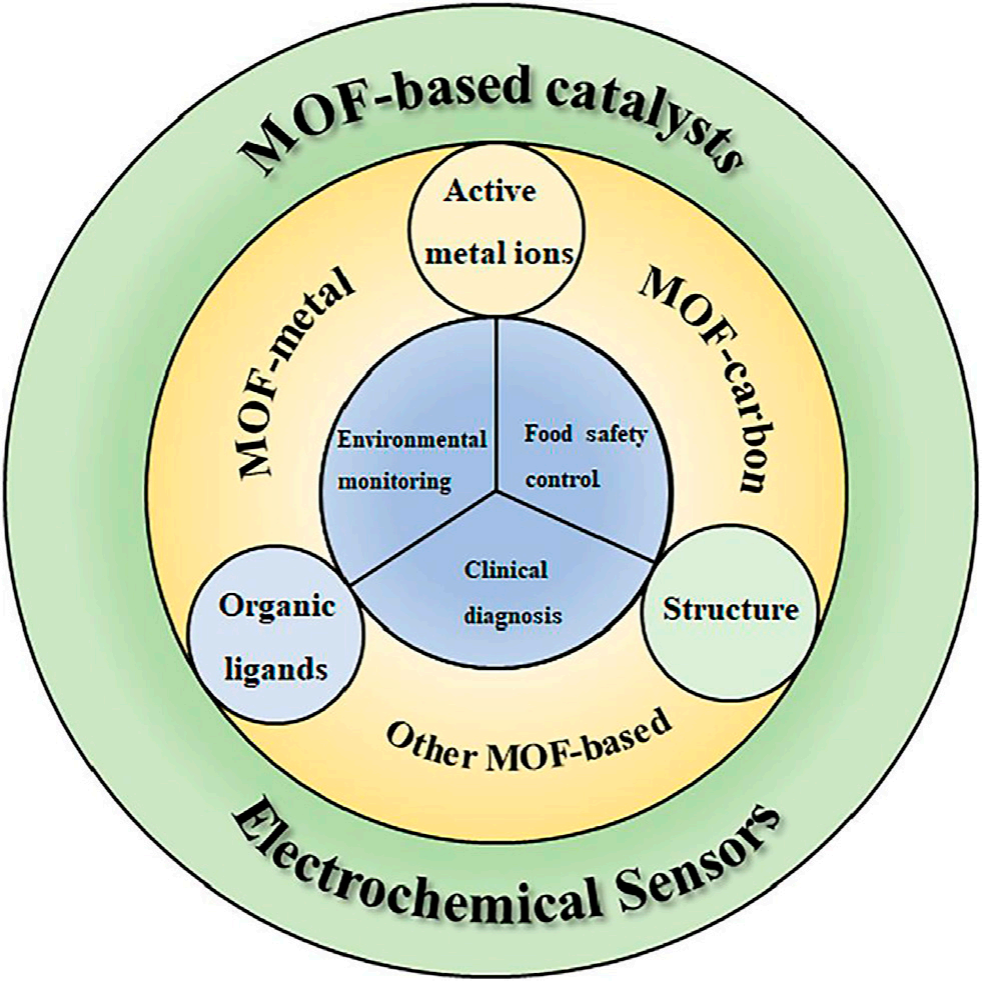
Mechanism and application of MOF-based catalysts as electrochemical sensors.

Developing efficient catalysts is vital for the application of electrochemical sensors. MOFs, with high porosity, large specific surface area, good conductivity, and biocompatibility, have been widely used in catalysis, adsorption, separation, and energy storage applications. In this review, based on the structure–activity–performance relationship of MOF-based catalysts, the mechanism of MOF-based catalysts as electrochemical sensors, including metal cations, synthetic ligands, and structure, is introduced. Then, the MOF-based composites are successively divided into metal-based, carbon-based, and other MOF-based composites. Furthermore, their application in environmental monitoring, food safety control, and clinical diagnosis is discussed (Figure 7). Recently, many efforts have been devoted to constructing high efficiency MOF-based catalysts. However, there still exist some challenges to achieve superior MOF-based catalysts for electrochemical sensors.

First, MOF materials with an excellent redox and catalytic activity in electrochemical sensors are still an urgent need. Due to the large surface area of MOFs, the nonspecific adsorption of coexisting substances will occur in the complex matrix, which will negatively affect the sensing performance. The fine regulation of the pore structure of MOFs can provide precision for the selective adsorption of the target. Furthermore, most MOFs with weak mechanical properties are unstable in water. Surface modification can be adopted to hydrophobic materials or introduce surface functional groups, which is a good method to improve the stability of MOFs in water.

Second, MOF-based catalysts for peroxidase-like enzymes have much lower catalytic activity than natural enzymes. Construction of MOF-based catalysts with higher surface area and richer active sites can alleviate this problem. Furthermore, the catalytic activity of MOFs can also be improved by reasonable selection of multivalent ligands and metal nodes. The weak binding force between the target analyte and MOFs affects the detection sensitivity. Surface functional group modification is an effective strategy to provide stronger binding sites for the adsorption of the target analyte. In addition to carbonizing MOFs and introducing highly conductive species to the host MOFs, *in situ* or posttreatment of doped conductive impurities to MOFs is an alternative to improve their conductivity, resulting in the improved performance.

In addition, during the synthetic and detection process, developing advanced observation techniques will be helpful to understand the structure–activity–performance relationship of MOF-based catalysts for electrochemical sensors. Moreover, the electrochemical sensors based on MOF catalysts still have limitations under laboratory conditions. Further research on MOF-based catalysts is needed to improve their electrochemical properties and pave the way for further application. With the development of nanoscience and biotechnology, it is believed that MOF-based electrochemical sensors will bring a broader development prospect in environmental, food safety, and clinical aspects.
